# An Entropy Approach to Multiple Sclerosis Identification

**DOI:** 10.3390/jpm12030398

**Published:** 2022-03-04

**Authors:** Gerardo Alfonso Perez, Javier Caballero Villarraso

**Affiliations:** 1 Department of Biochemistry and Molecular Biology, University of Cordoba, 14071 Cordoba, Spain; bc2cavij@uco.es; 2 Biochemical Laboratory, Reina Sofia University Hospital, 14004 Cordoba, Spain

**Keywords:** multiple sclerosis, DNA methylation, entropy

## Abstract

Multiple sclerosis (MS) is a relatively common neurodegenerative illness that frequently causes a large level of disability in patients. While its cause is not fully understood, it is likely due to a combination of genetic and environmental factors. Diagnosis of multiple sclerosis through a simple clinical examination might be challenging as the evolution of the illness varies significantly from patient to patient, with some patients experiencing long periods of remission. In this regard, having a quick and inexpensive tool to help identify the illness, such as DNA CpG (cytosine-phosphate-guanine) methylation, might be useful. In this paper, a technique is presented, based on the concept of Shannon Entropy, to select CpGs as inputs for non-linear classification algorithms. It will be shown that this approach generates accurate classifications that are a statistically significant improvement over using all the data available or randomly selecting the same number of CpGs. The analysis controlled for factors such as age, gender and smoking status of the patient. This approach managed to reduce the number of CpGs used while at the same time significantly increasing the accuracy.

## 1. Introduction

**Figure 1:**
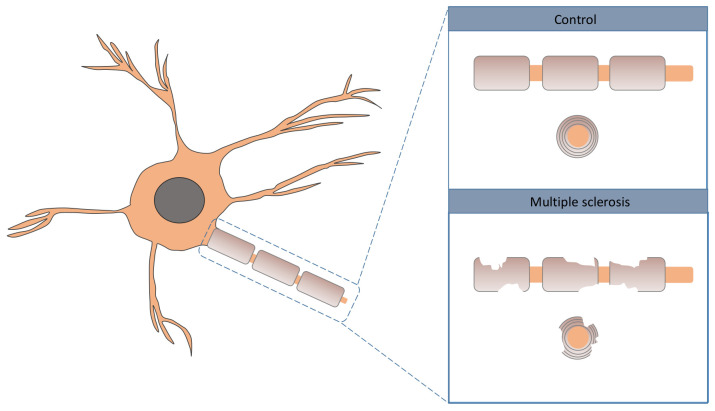
Graphical illustration of neurological damage in MS.

Multiple sclerosis (MS) is a chronic autoimmune illness affecting the brain and spinal cord associated with various degrees of disability. In MS, the immune system of the patient attacks the axons, more specifically, the myelin cover; see Figure 1 for a graphical illustration [1]. Inflammation is highlighted by some researchers as one of the drivers of neurodegeneration in MS [2,3,4]. The evolution of the illness varies greatly from patient to patient, with some individuals experiencing long periods of remissions due to mechanisms that are not yet well understood. The usual manifestation age of the illness is from 20 to 45 years old, but it can occasionally manifest at younger ages, even in children [5]. The causes of MS remain unclear, with a complex underlying combination of genetic and environmental factors the most likely cause [6,7,8,9,10].

There are some gender considerations to take into account, as the illness is more common in women than men in a 3:1 ratio (and in some countries like Sweden even 5:1). Some of the common symptoms of the illness include fatigue and numbness, typically in one side of the body [11,12]. Behavioral and cognition abnormalities are also common [13,14,15]. Currently there are many therapeutic approaches to control or stop the progression of the disease, but no curative treatment is available. However, a large amount of research has been generated regarding this disease. MS has a particularly high prevalence in some areas of Europe and the United States, particularly in northern regions [16].

**Figure 2:**
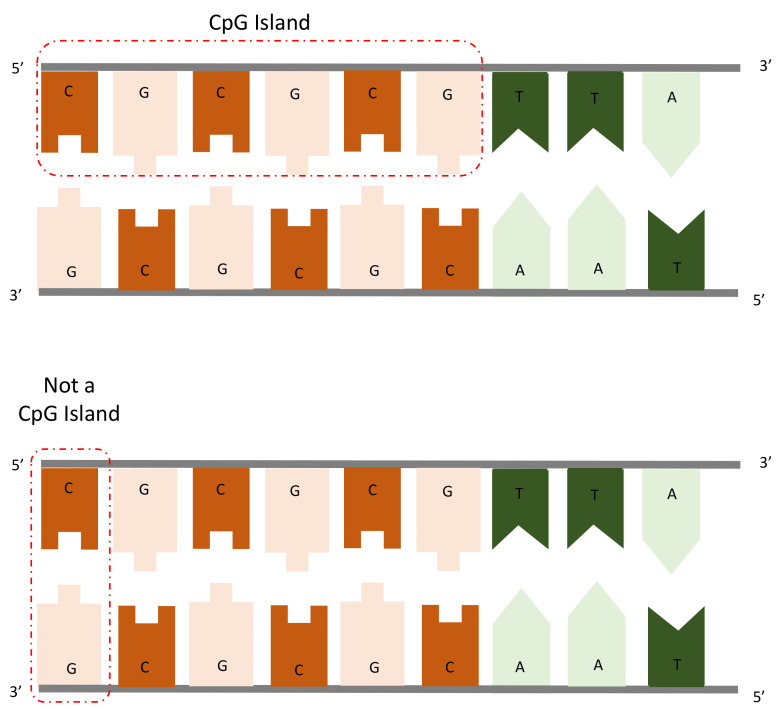
Illustration of CpG islands.

CpG DNA methylation data has been used to analyze neurodegenerative diseases such as Alzheimer’s [17,18,19,20] and Parkinson [21,22,23]. As can be seen in Figure 2, in the context of DNA methylation, CpG dinucleotide (or CpG) refers to cytosine followed by a guanine in the same DNA strand (typically 5*′* to 3*′*), not to be confused with cytosine and guanine pared in two complementary strands.

**Figure 3:**
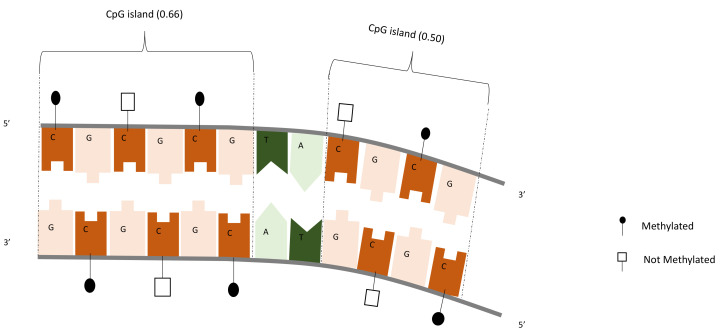
DNA methylation illustration.

Methylation is simply the addition of a methyl group at the 5-carbon (see Figure 3). DNA methylation has been extensively studied in the context of aging, with several biological clocks built using such types of data. Technological advances in recent years have made possible the analysis of DNA methylation levels on thousands of CpGs in a fast and reliable way. In practice, what is obtained is the percentage level of methylation with a value ranging from 0 to 1 (100% methylated). DNA methylation for cancer diagnostics has made significant progress in the last decades, including many seminal papers [24,25,26,27]. There is also a significant body of research covering diabetes [28,29,30,31,32].

DNA methylation has also been used in the context of multiple sclerosis [33,34]. Most of the existing literature on the topic tends to use linear approaches. In this paper, we have followed a non-linear approach, which is in principle more generic and encompassing than a linear approach. Machine learning techniques have been successfully used in multiple applications of different types of diseases [35,36,37,38]. More specifically, neural networks have been used as an algorithm for the identification of neurodegenerative illnesses, such as Alzheimer’s, using DNA methylation data as the input [39,40,41].

We applied the concept of Shannon Entropy in the context of DNA methylation applied to multiple sclerosis identification. As far as we are aware, this approach has not been followed before. Shannon Entropy is a concept initially developed in information theory, which attempts to quantify the amount of information contained in a certain set of data [42]. The precise mathematical definition of this concept will be introduced in the materials and methods section. It will be shown that using the concept of Shannon Entropy for CpG selection can generate accurate results.

### Motivation and Aims

Biomarkers are an increasingly important field, particularly when they can be analyzed using non or minimally invasive techniques. In this regard, blood is a particularly interesting tissue as it can be cheaply and quickly obtained from a patient causing only minimal discomfort. Blood has a significant advantage over other tissues such as brain matter, which is much harder to obtain. DNA methylation data can be accurately and rapidly analyzed using technologies such as the Illumina machines. Shannon Entropy is a concept frequently used in machine learning. The motivation to use this approach for data selection is in trying to find techniques that might reduce the dimensionality of the data. Shannon Entropy is one of the few concepts in the existing literature directly related to the amount of information contained in the data, which seems to be a reasonable starting point when trying to reduce the dimensionality of the data while maintaining as much information as possible.

The aim of this article is to develop techniques to identify DNA methylation signatures applicable for the identification of multiple sclerosis patients.

## 2. Materials and Methods

The DNA methylation data for each individual was stored in a vector $X^{i}$.

(1) $$X^{i}=\left\{\begin{array}{c} \;X_{1}^{i}\\ \;X_{2}^{i}\\ \vdots \\ \;X_{m}^{i} \end{array}\right\}$$

where *m* is the number of CpGs analyzed per patient. A numerical example would be:

(2) $$X^{2}=\left\{\begin{array}{c} \;0.211\\ \;0.723\\ \vdots \\ \;0.983 \end{array}\right\}$$

Which represents all the CpG information available for patient number 2. In this example, the methylation level in the first and second CpGs are 21.1% and 72.3%, respectively. As there is a large number of cases analyzed it is more convenient to group the data in a matrix form.

(3) $$X=\left(\begin{array}{cccc} \;X_{1}^{1} & X_{1}^{2} & \cdots & X_{1}^{n}\\ \;X_{2}^{1} & X_{2}^{2} & \cdots & X_{2}^{n}\\ \vdots & \vdots & \; & \vdots \\ \;X_{m}^{1} & X_{m}^{2} & \cdots & X_{m}^{n} \end{array}\right)$$

In this notation, there are *n* cases (including both patients and controls) with *m* CpGs associated with each case. The status of the individual analyzed (multiple sclerosis or control) was defined with a binary variable {0,1} stored in a target vector *T*, with the value 0 indicating a healthy control case and the value 1 indicating a patient with multiple sclerosis.

(4) T={0,1,0,⋯,1}

As there are *n* cases, there will be *n* entries for this vector. In this example, the first and third cases are control cases, and the second one a patient with MS. As a preliminary step, each CpG was individually linearly modeled against the classification vector *T* and only those with a *p*-value below 5% were included. The rest of the CpGs were discarded. The dimension of *X* was reduced from (n·m) to (n·l), where *l* is the number of CpGs with a *p*-value below 5%. *p*-value prefiltering was carried out in all the data. The Shannon Entropy (H) concept was then used to further filter the number of CpGs used. The Shannon Entropy approach step was carried out only for the training dataset. Shannon Entropy can be intuitively understood as the amount of information contained in some data and it is a concept borrowed from information theory. The mathematical expression for Shannon Entropy is as follows:

(5) $$H=-\sum_{i}P_{i}log_{2}\left(P_{i}\right)$$

This concept is typically applied in discrete mathematics. The probabilities can be estimated empirically. In simple terms, more entropy translates into more information contained. After the initial filtering, the absolute value of the Shannon Entropy was estimated for each CpG.

(6) $$H=\left\{\begin{array}{c} \;H_{1}\\ \;H_{2}\\ .\\ .\\ .\\ \;H_{l} \end{array}\right\}$$

Only CpGs with an entropy value $\left(H_{i}\right)$ bigger than certain predefined value $\left(H_{i}^{f}\right)$ were considered. All the other CpGs were excluded from the analysis. In this way we obtained $H^{*}$.

(7) $$H^{*}=\left\{\begin{array}{c} \;H_{1}^{*}\\ \;H_{2}^{*}\\ .\\ .\\ .\\ \;H_{q}^{*} \end{array}\right\}$$

In this notation q≤l. After selecting the CpGs, it is necessary to choose the classification algorithm that is used. A neural network with a hidden layer and an output layer was used. The hidden layer contained 50 artificial neurons, while the output layer contained a single artificial neuron. The 50 neurons in the hidden layer are of the sigmoid symmetric transfer function type. The neuron in the output layer is of the type sigmoid positive transfer function (both of these transfer functions are built-in in Matlab). All the neurons include a bias factor. The neural network was trained with the scaled conjugate backpropagation algorithm. Another four learning algorithms were tested (Levenberg–Marquardt, resilient backpropagation, one-step secant and gradient descent). As in the case of the transfer functions in the artificial neural networks, the learning algorithms are also built-in options in Matlab. Among all the learning algorithms, the best results were obtained using the scaled conjugate backpropagation approach. The data was divided into a training and a testing dataset. The testing dataset accounted for approximately 15% of the data. All the calculations were carried out in Matlab. Neural networks have been extensively used for modeling purposes and can accurately describe many complex underlying dynamics. An important step is to check that the classification error obtained using the above mentioned Shannon Entropy approach for CpG selection is more accurate than the one obtained when using the same number of randomly selected CpGs; in other words, controlling that the improvement in accuracy is not simply due to the reduction in the dimensionality of the data.

All the calculations were done in Matlab, the Shannon Entropy value was calculated using an existing Matlab function. The methylation data was analyzed using two decimals of precision in percentage terms. The analysis did not appear to be very sensitive to an increase to the third decimal place, but it started to have more impact thereafter (four or five decimal places in percentage terms). We believe that using two decimal places is a reasonable precision considering the likely accuracy of the experimental data.

A sensitivity analysis was also carried out. The underlying assumption was that CpGs with very little data variation would be less useful for classification purposes. In an extreme case, if the DNA methylation level for a given CpG was the same for all patients, then this information would not be useful for classification purposes. We did not assume that the CpGs with the most data variation (measured as the standard deviation) were necessarily the best choices, as other factors such as experimental noise (and potentially many others) can increase the variation of the data. However, it seemed reasonable to carry out a sensitivity analysis over reasonable values of the volatility of the DNA methylation data.

### Data

DNA methylation data for 279 individuals were obtained from the GEO database (publicly available data) with the accession code GSE 106648 [43]. The database contained both individuals with multiple sclerosis (140) as well as control individuals (139). The age range was from 16 to 66 years old, and there were 77 male individuals. There were more females than male patients. This is consistent with the observation that MS tends to be more common among females than males; 138 of the individuals in the dataset were smokers. Age, gender and smoking status (Table 1) were used as inputs in the model. As in the case of DNA methylation, these factors were allocated to their corresponding training or testing dataset.

The DNA methylation data [43] was obtained from peripheral blood tissue using the Illumina Human Methylation 450 Beach Chip. There were 485,512 CpG DNA methylation data per patient.

## 3. Results

**Figure 4:**
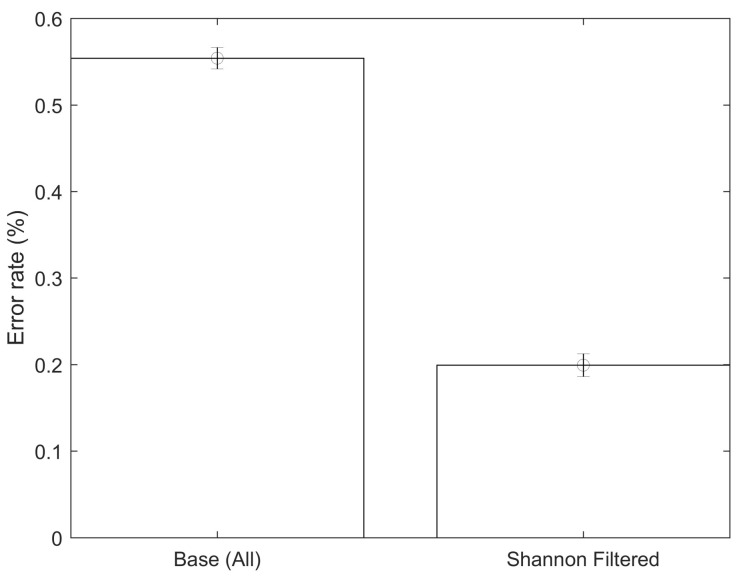
Error rate comparison between direct approach and Shannon Entropy filtered approach.

**Figure 5:**
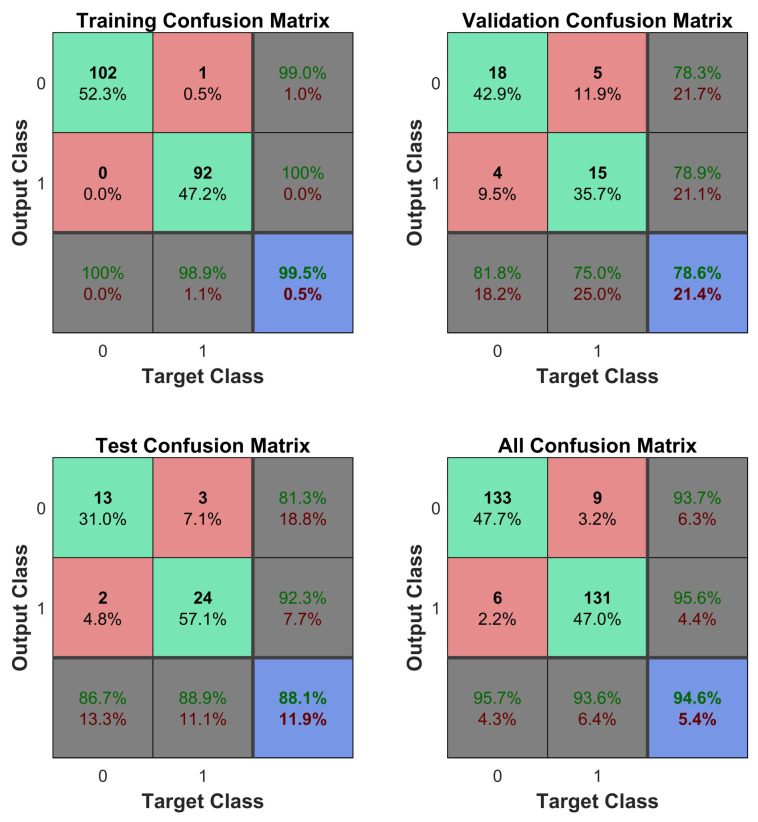
A sample confusion matrix (after *p*-value prefiltering and Shannon Entropy filtering).

**Figure 6:**
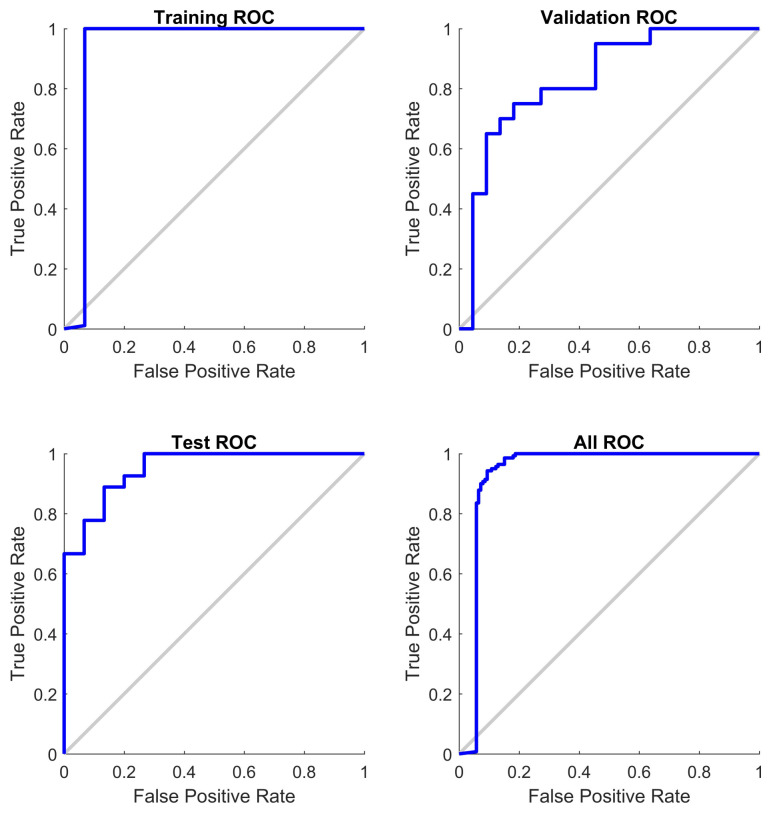
ROC (after *p*-value prefiltering and Shannon Entropy filtering).

As can be seen in Figure 4, the average classification error using all the available data with a *p*-value below 5% was 55.4%, while the error obtained when using only the CpGs with the top 10% Shannon Entropy values (9499 CpGs) was 19.93%, which is a statistically significant improvement. Equivalently, the proposed approach (using Shannon Entropy as a filter) generated a successful classification rate of approximately 80.07%, while the direct approach (using all the data) generated a successful classification rate of approximately 44.6%. The direct approach likely generates poor classifications due to the issue of local minima, which is likely improved by the introduced Shannon Entropy filtering. The model accuracy was substantially improved while at the same time reducing the amount of input data required in the mode. After the two steps (*p*-value filtering and Shannon Entropy filtering), the amount of CpGs was reduced by approximately 98% compared to the total initial data available. These results were obtained by dividing the data into training and testing datasets, with the testing dataset not used during the training phase. The testing dataset contained approximately 15% of the total data. Unless explicitly mentioned, all the results shown below refer to the testing dataset results. All the models controlled for age, gender and smoking status of the patients. As it can be seen in Table 2, the average sensitivity and specificity obtained were 78.3% and 81.8%, respectively. An example showing a confusion matrix and ROC can be seen in Figure 5 and Figure 6. 

In order to compare the results, two baseline values were obtained using the volatility (standard deviation) as an indicator. In the first baseline case, the top 2% most volatile CpGs were selected without any prefiltering (such as *p*-value). This was done in order to have a dimensionality comparable to the results obtained using the proposed approach (*p*-value prefiltering plus Shannon Entropy filtering). The classification success ratio using this technique was approximately 51.6%. A second base line level was obtained. In this case, *p*-value prefiltering was carried out followed by a selection of the most volatile CpGs. The threshold value for the volatility was selected in order to make the final dimension of the data, i.e., number of CpGs selected, approximately the same as the one obtained in the proposed approach (*p*-value plus Shannon filtering). The successful classification rate was 56.1%.

**Figure 7:**
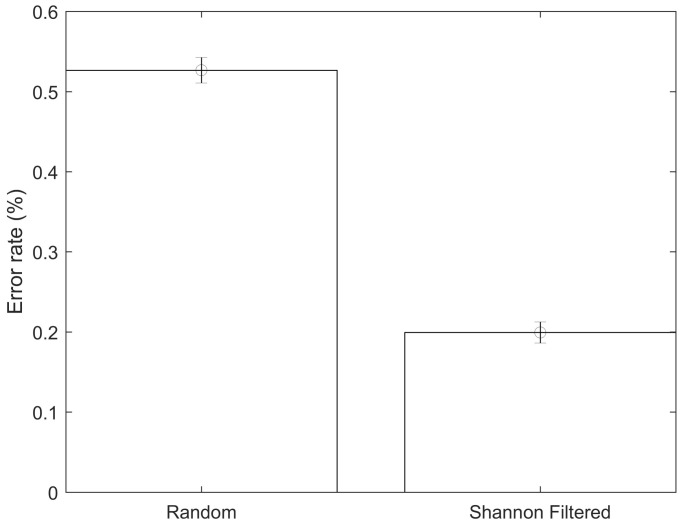
Error rate comparison between the Shannon Entropy filtered approach and random selection of the same size.

An important test to carry out is comparing the performance of the obtained CpGs by the Shannon Entropy approach (as inputs for the classification algorithm) to the results using a matrix of randomly selected CpGs. In this way, we account for the reduction in dimensionality of the data. Ten randomly selected sets of CpGs of the same size as the one obtained using the Shannon Entropy approach (9499) were selected. All the included CpGs in this random approach had *p*-values of less than 5%, i.e., this analysis was carried out after the initial linear filtering. Ten simulations were carried out for each of the ten different randomly selected sets of CpGs. The average value and the confidence interval can be seen in Figure 7. The Shannon Entropy approach generates classifications that are statistically significantly more accurate than a random selection of the same size.

**Figure 8:**
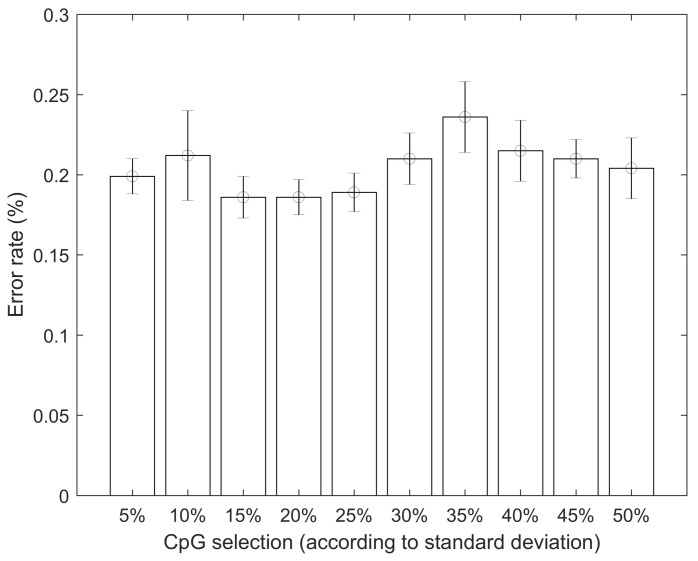
Sensitivity analysis according to the standard deviation of the value of the CpGs. Error rate as a function of the amount of CpGs selected according to their standard deviation.

As mentioned in the methods and materials section, a sensitivity analysis using the standard deviation of the DNA methylation data for each CpG was also carried out. In Figure 8, the results of selecting the CpGs with the highest volatility are shown. The range selected encompassed the top 5% to the top 50%, in 5% increments. For example, the first column shows the error rate (misclassifications) when using the top 5% of CpGs according to their standard deviation from the initial pool containing 9499 CpGs (after the initial filtering using Shannon Entropy filtering).

The intuition behind this approach is selecting CpGs with variation in the methylation values. As an extreme example, completely flat data (with standard deviation equal to zero) will arguably contain no value from a classification point of view. It is also acknowledged that some of that volatility might be caused by experimental and other sources of noise. The best results were obtained when using the top 15% most volatile CpGs with an average correct classification rate of 81.42%. However, the results were not statistically different (at a 5% significance) when compared with the results obtained by filtering for Shannon Entropy only (no filtering according to the standard deviation of the CpGs).

## 4. Discussion

An innovative approach is shown for the selection of DNA methylation CpGs to be used in non-linear classification models. This approach is based on the concept of Shannon Entropy, which it is an idea borrowed from the information theory field. Shannon Entropy, in simple terms, can be understood as a measure of the amount of information contained in a set of data. The overall data was first filtered, discarding the CpG with *p*-values above 5%. A quality pre-check of the data was also carried out, excluding CpGs with missing data. The analyzed dataset appeared to be of good quality with no major data issues. Using the two steps approach of *p*-value prefiltering followed by the proposed Shannon Entropy filtering, the dataset was reduced from an original size of approximately 485,512 to a final size of 9499 CpGs, which represents a 98% reduction. The classification analysis, distinguishing between control and multiple sclerosis patients, using the entire dataset, did not generate accurate results. The error rate when using the Shannon Entropy approach was 19.93% (80.07% correct classification), which is a statistically significant improvement over the base case. These error rates were obtained using artificial neural networks as the classification algorithm. All the analyses were carried out controlling for age, gender and smoking status of the patients. It was also tested if the increase in accuracy was due simply to the reduction in the dimensionality of the data. In order to do this, several random CpG configurations of the same size (9499 CpGs) as the one obtained using the Shannon Entropy approach were tested. Their average error rate was 52.66%, which is statistically significantly higher than the results obtained using the Shannon Entropy. This suggests that the Shannon Entropy approach might be a reasonable approach to select potential CpGs relevant for the classification analysis. This type of tool might become rather useful in the future, as the amount of CpGs analyzed per person increases and the computational costs increase accordingly. Another interesting analysis is controlling for the volatility, i.e., the standard deviation, of the CpGs. A sensitivity analysis was carried out in this regard by selecting CpGs according to their standard deviation (in buckets of 5%), i.e., top 5%, top 10%, and so on. When carrying out this type of analysis, there were some improvements in the average accuracy, but these improvements were not statistically significant.

These results were consistent with other articles that found a relationship between DNA methylation in other tissues such as the hippocampus [44]. Using blood as the selected tissue [43] is better suited for clinical purposes. Having a simple test, such as one based on DNA methylation data, which can be applied to many different diseases in a rapid and inexpensive way, can be useful. Multiple sclerosis is a relatively difficult illness to diagnose. Using only clinical symptoms and imaging, such as MRI, is frequently requested when the presence of illness is suspected. From a clinical point of view, it might be practical to have techniques, such as DNA methylation levels in the blood, which can be identified, with a reasonable level of accuracy, the presence of MS with a simple blood test. The physician can use the results from the blood-based biomarker combined with the clinical assessment to decide if it is necessary to carry out further tests, such as imaging.

A very interesting area of future research is the temporal evolution of the DNA methylation in multiple sclerosis, given the diverse evolution of the illness, particularly the long periods of remission experienced by some patients. Further research is necessary to determine feasibility, but it might be possible to use this type of approach for early detection. As more data becomes available, it might be possible to distinguish between different types of illness progression using DNA methylation data. It is possible that differentiating between the different types of evolution might help in targeting therapies in a more precise way.

## 5. Conclusions

Technical improvements are making possible the generation of large amounts of epigenetic data, such as DNA CpG methylation data, that can be used for the detection of several different types of illnesses, such as multiple sclerosis (MS). Multiple sclerosis is a complex illness with genetic and environmental factors, and importantly, an uncertain evolution with some patients experiencing long periods of remission. In this paper, we present a technique based on the Shannon Entropy concept for the selection of CpGs as inputs for MS identification using non-linear techniques such as artificial neural networks. It was shown that using the proposed approach, the number of CpGs used decreased while the accuracy of the classifications significantly improved. As more DNA methylation data becomes available, it is important to have techniques to efficiently filter these large amounts of information. In this regard, borrowing concepts like Shannon Entropy from other disciplines, such as information theory, might be an interesting approach. Having more data is likely beneficial but not all the new data will be helpful for analysis with a large percentage potentially adding noise. Therefore, it is important to develop techniques to further facilitate quantitative data analysis.

In the future, as more DNA CpG methylation data becomes available, it might be possible to extend this type of analysis in order to identify patients with different types of MS evolution. Currently, MS has no cure, but it is a field of intense research. It is possible that differentiating between the different types of evolution might help in targeting therapies in a more precise way, and this is a very appealing area of future research.

## Figures and Tables

**Table 1:** Basic descriptive information of the patients.

Description	Amount
Male	77
Female	202
Smokers	138
Non-smokers	141
Age	16, 77

**Table 2:** Average classification forecasting accuracy.

Accuracy Measure	Percentage
Average successful classification	80.1%
Sensitivity	78.3%
Specificity	81.8%

## Data Availability

The data is available in the GEO Database with accession code GSE 106648.
